# Spatially-integrated estimates of net ecosystem exchange and methane fluxes from Canadian peatlands

**DOI:** 10.1186/s13021-018-0105-5

**Published:** 2018-09-20

**Authors:** K. L. Webster, J. S. Bhatti, D. K. Thompson, S. A. Nelson, C. H. Shaw, K. A. Bona, S. L. Hayne, W. A. Kurz

**Affiliations:** 1 Natural Resources Canada, Canadian Forest Service, Great Lakes Forestry Centre, 1219 Queen St. E, Sault Ste. Marie, ON P6A 2E5 Canada; 2 Natural Resources Canada, Canadian Forest Service, Northern Forestry Centre, 5320 122 Street NW, Edmonton, AB T6H 3S5 Canada; 3 Environment and Climate Change Canada, Science and Technology Branch, 351 St. Joseph Boulevard, Gatineau, QC K1A 0H3 Canada; 4 Natural Resources Canada, Canadian Forest Service, Pacific Forestry Centre, 506 Burnside Road W, Victoria, BC V8Z 1M5 Canada

**Keywords:** Peatlands, Net ecosystem exchange, Methane, Peatland map, Spatial integration, National estimates

## Abstract

**Background:**

Peatlands are an important component of Canada’s landscape, however there is little information on their national-scale net emissions of carbon dioxide [Net Ecosystem Exchange (NEE)] and methane (CH_4_). This study compiled results for peatland NEE and CH_4_ emissions from chamber and eddy covariance studies across Canada. The data were summarized by bog, poor fen and rich-intermediate fen categories for the seven major peatland containing terrestrial ecozones (Atlantic Maritime, Mixedwood Plains, Boreal Shield, Boreal Plains, Hudson Plains, Taiga Shield, Taiga Plains) that comprise > 96% of all peatlands nationally. Reports of multiple years of data from a single site were averaged and different microforms (e.g., hummock or hollow) within these peatland types were kept separate. A new peatlands map was created from forest composition and structure information that distinguishes bog from rich and poor fen. National Forest Inventory k-NN forest structure maps, bioclimatic variables (mean diurnal range and seasonality of temperatures) and ground surface slope were used to construct the new map. The Earth Observation for Sustainable Development map of wetlands was used to identify open peatlands with minor tree cover.

**Results:**

The new map was combined with averages of observed NEE and CH_4_ emissions to estimate a growing season integrated NEE (± SE) at − 108.8 (± 41.3) Mt CO_2_ season^−1^ and CH_4_ emission at 4.1 (± 1.5) Mt CH_4_ season^−1^ for the seven ecozones. Converting CH_4_ to CO_2_ equivalent (CO_2_e; Global Warming Potential of 25 over 100 years) resulted in a total net sink of − 7.0 (± 77.6) Mt CO_2_e season^−1^ for Canada. Boreal Plains peatlands contributed most to the NEE sink due to high CO_2_ uptake rates and large peatland areas, while Boreal Shield peatlands contributed most to CH_4_ emissions due to moderate emission rates and large peatland areas. Assuming a winter CO_2_ emission of 0.9 g CO_2_ m^−2^ day^−1^ creates an annual CO_2_ source (24.2 Mt CO_2_ year^−1^) and assuming a winter CH_4_ emission of 7 mg CH_4_ m^−2^ day^−1^ inflates the total net source to 151.8 Mt CO_2_e year^−1^.

**Conclusions:**

This analysis improves upon previous basic, aspatial estimates and discusses the potential sources of the high uncertainty in spatially integrated fluxes, indicating a need for continued monitoring and refined maps of peatland distribution for national carbon and greenhouse gas flux estimation.

**Electronic supplementary material:**

The online version of this article (10.1186/s13021-018-0105-5) contains supplementary material, which is available to authorized users.

## Background

Canada is second only to Russia in peatland area [1]; this extensive area provides many important ecosystem services such as water storage, wildlife habitat, and carbon (C) sequestration [2]. Across Canada, peatlands are estimated to store 103–184 Pg C [3]. Tarnocai [4] estimated the organic C pool of Canadian peatlands to be 147 Pg, of which 67% occurs in the Boreal and 30% in the Subarctic peatland regions [5]. Canadian peatland soils store 60% more C than that stored in forest biomass and soils [6]. The C stored in peatlands represents the balance between above and belowground net primary production and decomposition in both the upper, periodically aerobic (acrotelm) peat layer and the underlying, anaerobic (catotelm) peat layer. In general, peatlands have much lower productivity than other natural ecosystems and peat accumulation is controlled by cool, wet conditions that limit decomposition [7]. The importance of peatlands in the C balance of Canada and the globe has been recognized for decades [8] but we still have a limited understanding of the spatial distribution of peatlands relative to forest ecosystems and their net greenhouse gas (GHG) balance at a national scale, despite a relatively sophisticated understanding and modelling capacity at the plot level (e.g., [9]). This knowledge gap in the national peatland GHG balance must be addressed to satisfy growing international pressure for better GHG estimation and reporting of organic soils on managed lands [e.g., Intergovernmental Panel on Climate Change (IPCC) Supplement to the 2006 Guidelines for National Greenhouse Gas Inventories: Wetlands (Wetlands Supplement—IPCC [10]).

The two existing estimates of national GHG emissions from peatlands in Canada [6, 11] used the Peatlands of Canada database [12, 13] and a single net C accumulation or CH_4_ emission factor for all peatlands. The net C accumulation (or sequestered CO_2_–C) rate for both was assumed to equal the estimate of the average long-term apparent rate of C accumulation (LORCA) over the last six to eight thousand years of 20–30 g C m^−2^ year^−1^ (i.e., uptake of 73–110 g CO_2_ m^−2^ year^−1^) [8, 14–17]. The CH_4_ emission rate was determined by expert opinion to be 0.8 g CH_4_ m^−2^ year^−1^ (Tarnocai, pers comm) in Kurz et al. [11] and to be 2 g CH_4_ m^−2^ year^−1^ from an average of published studies [18] for Roulet [6]. Multiplying the peatland area from the Peatlands of Canada database (1.1 × 10^6^ km^2^) by emissions, Kurz et al. [11] estimated for the year 1986 a Canada-wide peatland net C sink of 96.0 Mt CO_2_ year^−1^ and a CH_4_ release of 0.75 Mt CH_4_ year^−1^. Roulet [6] arrived at a similar Canada-wide estimate for a net C sink of 91.6–135.6 Mt CO_2_ year^−1^, but a higher estimate of CH_4_ release of 2–5 Mt CH_4_ year^−1^ [6, 18].

The Peatlands of Canada Map (PCM; [12, 13]) was developed using the Soil Landscapes of Canada (SLC) database [19], which contains soil information for each polygon based on reconnaissance soil survey. It includes other soil types that occupy a small area, such as Folisols which are upland organic (folic) materials, generally of forest origin, that are greater than 40 cm in thickness [20]. This polygon-mapping product depicting proportional peatland areas of differing classes in Canada at the 1:1 million scale has been available for some time (e.g., [21, 22]) and was based on air photo interpretation and manual delineation.

The lack of high-resolution functional peatland maps has been identified as a barrier to proper regional estimates of peatland C exchange [23]. The PCM does not identify key functional differences in peatland types. For example, fens occur along a nutrient gradient [7] with rich-intermediate fens having different C dynamics than poor fens (e.g., [24, 25]), yet these categories are not differentiated in the map. Another key functional difference not included in the PCM is the degree of tree cover (i.e., open, treed vs. forested). The importance of canopy cover in net CO_2_ exchange is evident in the close to doubling of annual NEE in bog and fen systems with higher leaf area index [26]. Even within rich fens, a higher canopy cover is indicative of drier surface conditions and therefore smaller CH_4_ emissions [27].

**Fig. 1:**
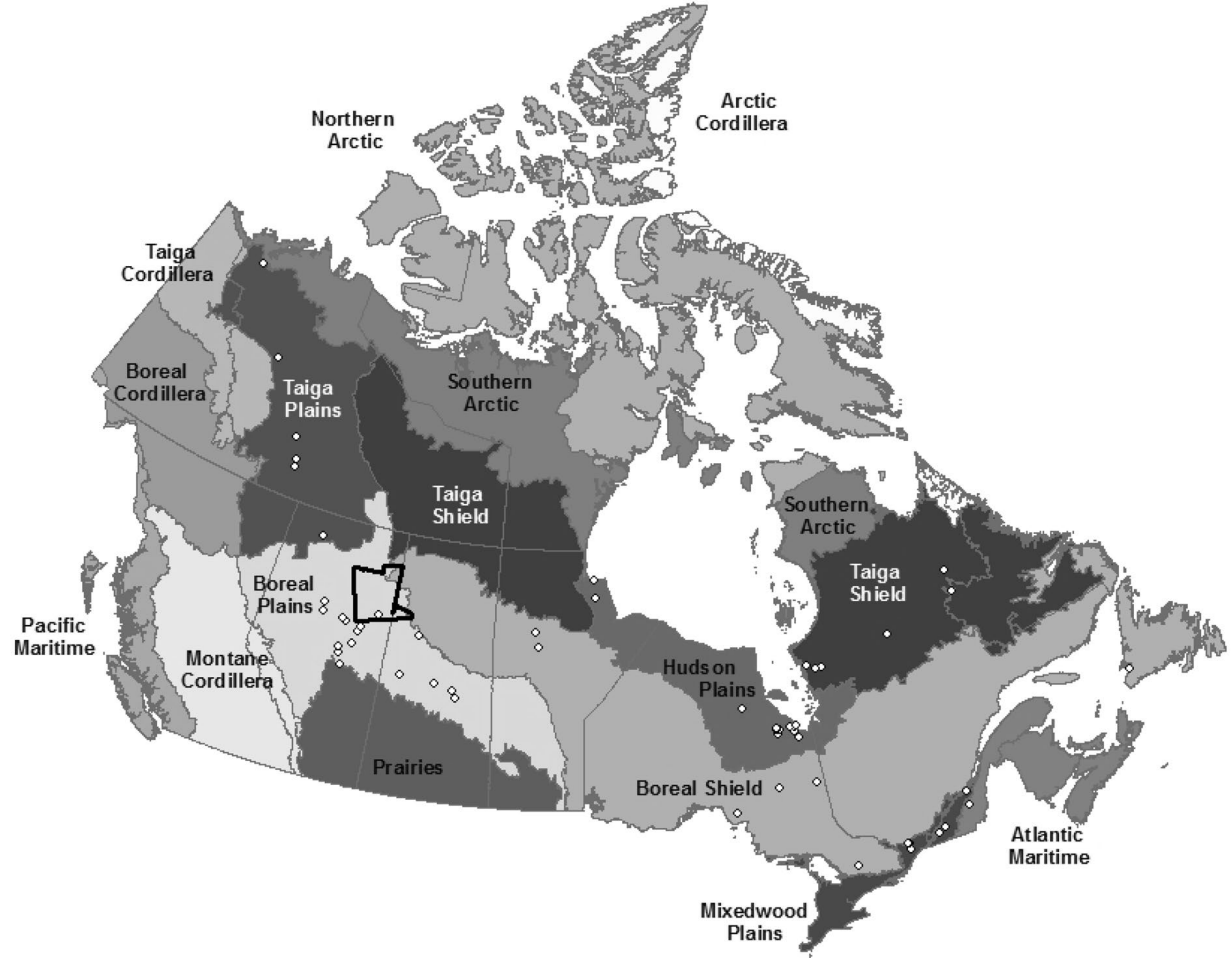
Map of ecozones [69] with locations of sources of data for net ecosystem exchange and methane emissions indicated by individual points. The location of the case study area with the Ducks Unlimited ground validation points for new peatland map is outlined in black

In the past 20–30 years there has been a large increase in the number of plot-based studies measuring NEE and CH_4_ emission rates across different peatland types and in different ecozones in response to the need for better understanding of the controls on C cycling within peatlands (Fig. 1). These studies range from single measurements at a single site over a short time period using chamber measurements, to short-term chamber studies done at many sites (e.g., Northern Wetland Study [NOWES; [28]) to multi-year studies using eddy covariance (EC) techniques across a network of sites (e.g., Fluxnet Canada [29], Boreal Ecosystem-Atmosphere Study [BOREAS; [30]). These studies have been focused on a range of scientific questions including: process-based hydrochemical controls on gas fluxes, local scale influence of microforms (e.g., hummocks and hollows), experimental manipulations of temperature and water table, and intra- and inter-annual variability in C emissions. The results of these studies have shown that environmental factors such as water table [31–34], soil temperature [35, 36], mineral nutrient and soil salinity [37–39], and vegetation biomass and type [36, 40] likely have strong controlling effects on temporal and spatial variability in NEE and CH_4_ emissions from peatland ecosystems.

The purpose of this study is to: (1) synthesize available estimates of NEE and CH_4_ for bogs, poor fens, and rich-intermediate fens for each of seven major peatland containing ecozones in Canada, (2) create a new 250 m resolution, raster-based peatland map synthesized from existing national landcover and forest structure maps and compare it to the polygon-based PCM [13], (3) provide new national estimates of CH_4_ and NEE emissions and the net greenhouse gas balance using the synthesized data combined with the new map, and for comparison combined with the PCM and, (4) investigate potential bioclimatic drivers of emissions of CH_4_ and NEE.

## Methods

### NEE and CH_4_ emissions

The literature was surveyed and researchers contacted to find studies (Fig. 1) conducted within Canada that measured NEE and CH_4_ emissions (Additional file 1). A total of 52 papers and several unpublished data sets were assessed, resulting in 66 values of NEE and 157 values of CH_4_ fluxes. These values were synthesized from many more measurements. For sites with multiple years of measurement, the most recent study that reported the most measurement dates was used and averages of plots and/or microforms were calculated across years. If values for microforms were reported separately, they were used in this analysis as separate values in order to capture the variability in fluxes from the peatland. If provided, for each data source information, was recorded on: year of study, months of measurement, ecological type, microtopography, pH, measurement method (chamber or EC), dominant plant functional types, average hourly, daily, seasonal and annual NEE and CH_4_ flux. For the purposes of this paper peatland types were classified as rich-intermediate fen, poor fen or bog either by using the classification provided in the study or, if none was provided, classification was based on data provided for pH or dominant plant functional types. Fens were defined by the presences of geogenous water, with rich-intermediate fens having high pH (≥ 5.5) and dominated by true mosses, while poor fens have a lower pH (< 5.5) and are dominated by peat mosses [7]. Bogs are ombrotrophic, having drier surface conditions, and are dominated by oligotrophic *Sphagnum* species of mosses [7]. We recognize that swamps and marshes are also important wetlands types, and that in some regions of Canada these wetland types contain thick organic layers which may meet the Canadian peatland definition [21]. Permafrost is also an important feature within peatlands, affecting GHG dynamics. However swamps and marshes and permafrost peatlands they are not included in this analysis, but will be the focus of future work.

All surveyed emission values were converted to average daily growing season NEE and CH_4_ values as a base unit of comparison. We recognize that international reporting uses annual emissions, but for the purposes of this study daily is used to control for vast differences in growing season length among the different studies, and to incorporate studies that only reported daily fluxes. For studies where only growing season (“seasonal”) estimates of NEE and CH_4_ emissions were provided, daily estimates were calculated from seasonal estimates by dividing by length of growing season (GS; i.e., the number of days between the period that mean daily temperature was greater than or equal to 5 °C for five consecutive days beginning March 1st and the minimum temperature less than − 2 °C beginning August 1st [41]), extracted for each study location from 300 arc-second (~ 10 km) resolution climate surfaces of McKenney et al. [42]. Daily NEE (g CO_2_ m^−2^ day^−1^) is defined as (Eq. 1);

1 $$Daily \, NEE = \frac{Seasonal \, NEE}{GS} \times 5$$

A scaling factor of five was determined using studies from the literature that reported both daily and seasonal NEE. The scaling factor is required to make the relationship between observed daily NEE and daily calculated from seasonal NEE 1:1 (r^2^ = 0.62, p = 0.001). The scaling factor helps to account for NEE that occurs in the shoulder seasons therefore avoiding under prediction. When only annual NEE values were reported, seasonal NEE was determined using an assumed rate of 1.0 g CO_2_ m^−2^ day^−1^ [based on an average of winter CO_2_ emission rates reported from the literature (Table 1)] for the non-growing season period (i.e., 365-GS) before converting to a daily rate as explained above.

**Table 1:** Non-growing season CO_2_ emissions (g CO_2_ m^−2^ day^−1^) reported from different studies

Reference	Non-growing season CO_2_ emission (g CO_2_ m^−2^ day^−1^)
Roehm and Roulet [58]	1.0
Lafleur et al. [56]	1.0
Strack et al. [83] (hummock)	0.4
Strack et al. [83] (lawn)	0.3
Strack et al. [83] (hollow)	0.7
Strack and Zuback [90]	0.9
Trudeau et al. [60] (hollow)	0.2
Trudeau et al. [60] (hummock)	2.7
Trudeau et al. [60] (lawn)	0.7
Wang et al. [91]	1.5
Average	0.9

No scaling factor was required for converting seasonal CH_4_ emissions (g CH_4_ m^−2^ season^−1^) to daily rates (mg CH_4_ m^−2^ day^−1^) (Eq. 2) as the relationship between observed daily CH_4_ and daily calculated from seasonal CH_4_ was 1:1 (r^2^ = 0.72, p < 0.001);

2 $$Daily\,CH_{4} = \frac{{Seasonal\,CH_{4} }}{GS} \times 1000$$

When only annual CH_4_ values were reported, seasonal CH_4_ was determined using an assumed rate of 7 mg CH_4_ m^−2^ day^−1^ [based on an average of winter CH_4_ emission rates reported from the literature (Table 2)] in a similar manner as describe for NEE.

**Table 2:** Non-growing season CH_4_ emissions (g CH_4_ m^−2^ day^−1^) reported from different studies

Reference	Non-growing season CH_4_ emission (mg CH_4_ m^−2^ day^−1^)
Strack et al. [84] (hummock)	19.4
Strack et al. [84] (lawn)	17.0
Strack et al. [84] (hollow)	0.6
Pelletier et al. [57] (hummock)	2.5
Pelletier et al. [57] (hummock with shrubs)	1.9
Pelletier et al. [57] (hollow)	4.7
Pelletier et al. [57] (sedges and vascular)	4.6
Trudeau et al. [92]	2.7
Strack and Zuback [90]	8.1
Average	6.7

Default (Tier 1) emission factors for annual peatland CO_2_ and CH_4_ emissions presented in the IPCC Wetlands Supplement were developed by assuming non-growing season emissions equaled 15% of growing season emissions (15% of ecosystem respiration for CO_2_). However, growing season ecosystem respiration data was not available for most of the Canadian studies. For the studies that did have ecosystem respiration, there was a good relationship (r^2^ = 0.87, p < 0.001) between annual NEE calculated where the non-growing season emission was estimated as 15% of ecosystem respiration, and where it was estimated using 0.9 g CO_2_ m^−2^ day^−1^ for non-growing season days. To be consistent with the method for NEE the constant daily CH_4_ rate was used, and there was also a good relationship between annual CH_4_ estimated by calculating the non-growing season emission as 15% of the seasonal CH_4_ emission, and calculating it using 7 mg CH_4_ m^−2^ day^−1^ for non-growing season days (r^2^ = 1.0, p < 0.001).

Global warming potential (GWP) was calculated by converting CH_4_ to CO_2_ equivalents (CO_2_e) using a factor of 25 over a 100 years time horizon and then adding the estimated CO_2_e from CH_4_ to NEE, acknowledging the GWP approach is not the full story in considering the net radiative forcing of peatland ecosystems over longer time scales. Differences among peatland types and regions were assessed using ANOVA or ANOVA on ranks (if normality criteria not met) in SigmaPlot v12.0 [43].

### New peatlands map

**Fig. 2:**
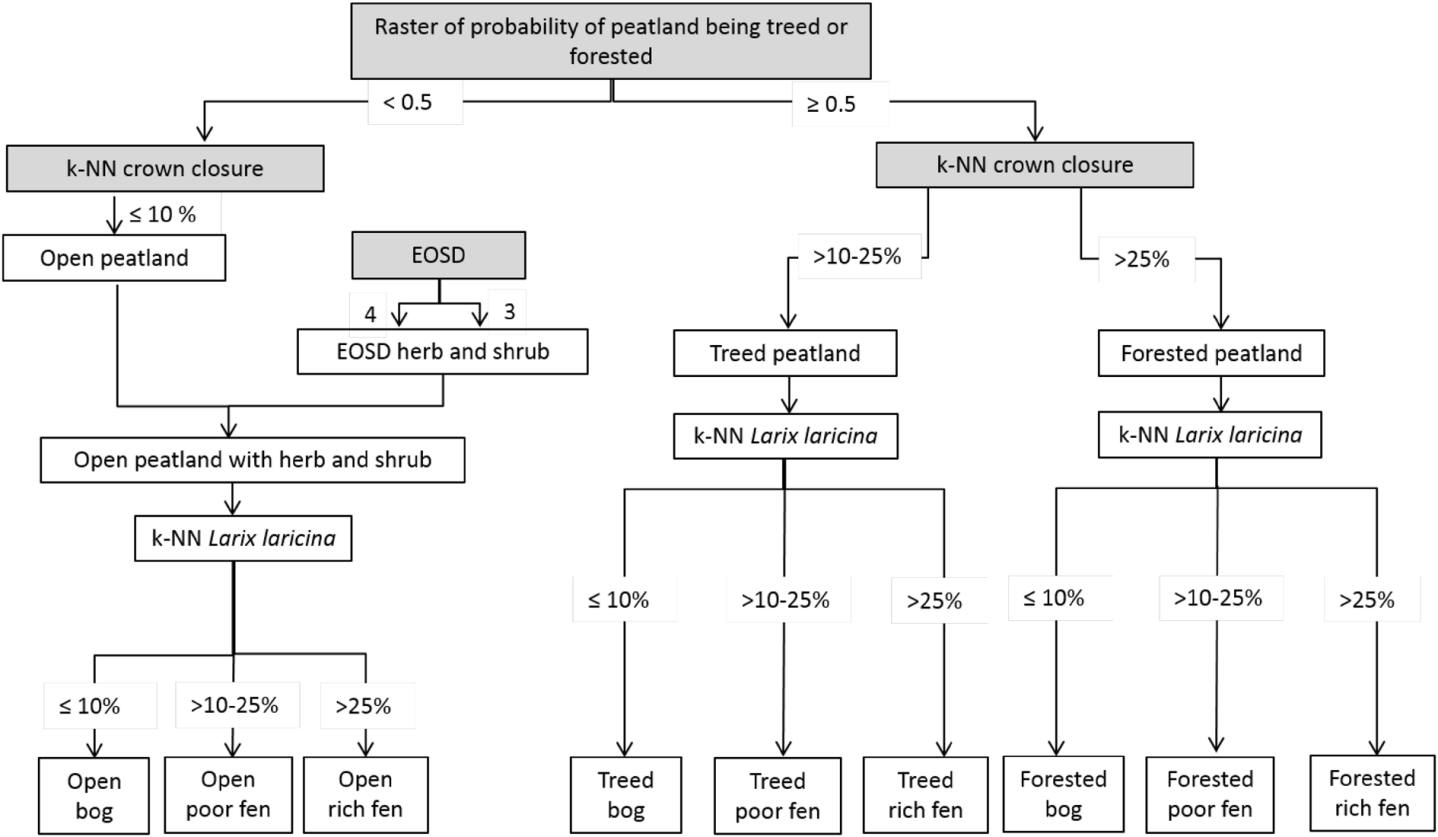
Flow chart of raster layers used to create the new peatland map. The raster of the probability of a peatland being treed or forested is the map from [45]. K-NN corresponds to the National Forest Inventory k-NN mapping product from Beaudoin et al. [46] and EOSD corresponds to the Earth Observation for Sustainable Development of Forests (EOSD) map of wetlands from Wulder et al. [49]

The new peatlands map (250 m pixel resolution) contains nine peatland categories (open, treed, and forested for each of the peatland types of rich-intermediate fen, poor fen, and bog) to accommodate future spatial peatland C modelling with the Canadian Model for Peatlands [44]. The updated peatlands map was created based on the forested peatlands map created by Thompson et al. [45] by combining forest composition and structure information with the National Forest Inventory (NFI) k-NN mapping product from Beaudoin et al. [46], bioclimatic variables (mean diurnal range and seasonality of temperatures) and ground surface slope. Of the two models presented in Thompson et al. [45], the raster product using the boosted regression tree method [47] was used, and a threshold model output of 0.5 (Fig. 2) was set for the classification of treed and forested peatlands. The Canadian National Hydro Network [48], vector layers of open water bodies was used to mask out water bodies. Since the k-NN map includes only peatlands with significant tree cover, the Earth Observation for Sustainable Development of Forests (EOSD; [49]) map of wetlands was used to identify open (both shrub, herb, or moss cover only) peatlands with only minor (< 10% canopy closure) tree cover. The original 30 m resolution EOSD product was resampled to 250 m using the majority resampling technique and reprojected to the k-NN grid. The NFI k-NN layer for tamarack [*Larix laricina* (Du Roi) K. Koch] was used to further differentiate bogs, poor fens, and rich fens (Fig. 2), as the proportion of tree cover as tamarack increases from zero in ombrotrophic bogs to 100% in rich fens [50, 51]. Bogs were mapped in areas where the proportion of tree biomass as tamarack was less than 10%, though in true ombrotrophic bogs the proportion is close to zero [50]. This larger margin of tamarack takes into account the uncertainty in the prediction of tamarack in the k-NN dataset [45]. Accordingly, pixels were designated as poor fens when tamarack content was 10–25%, and rich fens when tamarack cover was over 25%. For the C flux analysis presented here the nine peatland categories are reduced to three (rich-intermediate fen, poor fen, and bog) since there was insufficient information given in the surveyed GHG studies to accurately classify them in more detail (e.g., vegetation cover).

Evaluating the accuracy of the new peatlands map is problematic given the lack of detailed ground-truthed peatland maps containing all nine peatland types. Several different approaches were used to evaluate the map accuracy. First, the total area of peatlands estimate by the PCM and the new peatlands map were compared. Then the new peatland map was overlain by the PCM polygons to calculated percentages of peatland types (collapsed to only bog and fen categories) based on the new peatland map to compare with the same percentages from the PCM for each polygon. The new peatlands map was also qualitatively evaluated by cross-referencing the new map to the geographic locations of the reported CO_2_ and CH_4_ flux studies. Finally, for a small region in northern Alberta, ground validation points collected by Ducks Unlimited Canada (DUC) were used to evaluate accuracy in predicting peatland categories.

### Scaling emissions

To spatially scale site NEE and CH_4_ emissions for the seven major peatland containing ecozones within Canada average daily NEE and CH_4_ emissions for each ecozone were converted to seasonal NEE and CH_4_ emissions using an average GS for each ecozone, by reversing the method described in “NEE and CH4 emissions” section. Seasonal integration was chosen since many studies, with the possible exception of eddy covariance studies, are only done for the growing season. Annual integration is also presented using a constant daily emission of CO_2_ and CH_4_ for the non-growing season, acknowledging there is incomplete knowledge and high uncertainty regarding winter emissions.

Two spatially-integrated estimates were calculated. For the first estimate, hereafter referred to as the Peatlands of Canada approach, seasonal ecozone averages of NEE and CH_4_ emissions for bog and fen (average of poor and rich-intermediate fen) were applied as emission factors to peatland areas from the polygon-based PCM for the seven major peatland-containing ecozones. Areas of different peatland types were calculated by multiplying the percent peatland type by polygon area and summed to provide estimates for each ecozone. Where there were no NEE and CH_4_ emissions for a peatland type and ecozone combination or no standard error (SE) could be calculated (i.e., n = 1), the emission or SE for that peatland type in the most similar ecozone was used. For the second estimate, hereafter referred to as the new peatlands map approach, seasonal ecozone averages of NEE and CH_4_ emissions for bog, poor fen and rich-intermediate fen for each of the seven major peatland-containing ecozones were applied as emission factors to peatland areas from a newly created raster-based peatland map. Poor fens and rich-intermediate fens were treated separately in the second estimate because they are differentiated in the new mapping product, but not in the PCM.

### Potential climatic drivers of peatland NEE and CH_4_ emissions

A tree regression approach was used to examine relationships between climate and peatland NEE and CH_4_ emissions from the studies, using a suite of bioclimatic factors extrapolated for each of the study locations from a national climate surface interpolation model [300 arc-second (~ 10 km)] [42]. The suite of bioclimatic factors included: mean temperature, total precipitation and length of growing season for annual and warmest quarter of the year, mean diurnal range [mean of all the weekly diurnal temperature ranges (maximum–minimum)], isothermality (mean diurnal range divided by the annual temperature range), temperature seasonality (temperature coefficient of variation), annual temperature range, precipitation seasonality (precipitation coefficient of variation), start day of growing season (Julian day of mean daily temperature that was greater than or equal to 5 °C for five consecutive days beginning March 1) and end day of growing season (Julian day of minimum temperature less than − 2 °C beginning August 1st [41]). The tree regression was run using Rpart library within R [52] using the bioclimatic factors along with peatland type as the predictor variables and daily NEE or CH_4_ emissions as the response variables. The tree regression used the ‘anova’ method and default values for rpart.control arguments. In the tree regression, the coefficient of determination (r^2^) is calculated as 1-relative error.

## Results

### NEE and CH_4_ emissions

Summarizing the studies examined, most of the NEE measurements were from bogs and the least from poor fens (Table 3). For CH_4_ emissions, both bog and rich-intermediate fens were well represented, with fewer studies reporting on poor fens (Table 3). Studies existed for at least one peatland type within each of the seven ecozones (Table 3). The Atlantic Maritime was the least represented, with only NEE and CH_4_ values for bogs. The Boreal Plains, Boreal Shield and Mixedwood Plains were best represented having studies for NEE and CH_4_ for all peatland types occurring within the ecozone. Hudson Plains was missing poor fen NEE and CH_4_, Taiga Shield was missing bog and rich-intermediate fen for NEE and Taiga Plains was missing poor fen for NEE.

**Table 3:** Average and standard error of the mean (SE) of daily growing season net ecosystem exchange (NEE) and methane (CH_4_) emissions for each of the major peatland-containing ecozones [69]

(A) Fen categories combined for use with Peatlands of Canada Map												
Ecozone	NEE (g CO_2_ m^−2^ day^−1^)						CH_4_ (mg CH_4_ m^−2^ day^−1^)					
	Bog			Fen			Bog			Fen		
	n	Avg	SE	n	Avg	SE	n	Avg	SE	n	Avg	SE
Atlantic Maritime	5	− 0.7	5.2	0	− *1.3*	*2.8*	7	40.8	7.0	0	*65.8*	*8.4*
Boreal Plains	11	− 8.6	2.7	6	− 11.2	1.2	3	2.2	2.1	12	78.8	23.6
Boreal Shield	13	− 4.4	2.0	5	− 0.2	1.4	28	33.0	8.4	31	39.6	7.8
Hudson Plains	5	− 3.8	1.4	7	− 0.9	2.2	15	28.8	7.8	17	17.1	6.0
Mixedwood Plains	2	− 3.1	0.1	4	− 1.3	2.8	3	7.0	6.5	3	65.8	8.4
Taiga Plains	1	− 1.3	*2.7*	4	− 5.8	3.2	4	124.2	54.9	4	63.8	36.5
Taiga Shield	0	− *1.3*	*2.7*	3	0.3	0.5	2	27.0	21.0	28	34.3	7.4
Total	37	− 4.9	1.3	29	− 3.5	1.1	62	35.8	6.2	95	40.8	5.1

(B) Fen categories separated for use with new peatlands map																		
Ecozone	NEE (g CO_2_ m^−2^ day^−1^)									CH_4_ (mg CH_4_ m^−2^ day^−1^)								
	Bog			Poor fen			Rich-intermediate			Bog			Poor fen			Rich-intermediate		
	n	Avg	SE	n	Avg	SE	n	Avg	SE	n	Avg	SE	n	Avg	SE	n	Avg	SE
Atlantic Maritime	5	− 0.7	5.2	0	− *1.3*	*2.8*	0	− *0.9*	*2.2*	7	40.8	7.0	0	*65.8*	*8.4*	0	*17.1*	*6.0*
Boreal Plains	11	− 8.6	2.7	1	− 12.5	*0.5*	5	− 10.2	1.1	3	2.2	2.1	1	0.9	*9.4*	11	85.8	24.7
Boreal Shield	13	− 5.5	2.1	1	0.4	*0.5*	4	− 0.3	1.8	28	33.0	8.4	12	34.2	9.4	19	43.0	11.4
Hudson Plains	5	− 5.4	1.7	0	− *1.3*	*2.8*	7	− 0.9	2.2	15	28.8	7.8	0	*34.2*	*9.4*	17	17.1	6.0
Mixedwood Plains	2	− 7.6	2.1	4	− 1.3	2.8	0	*Not mapped*		3	3	7.0	3	65.8	8.4	0	*Not mapped*	
Taiga Plains	1	− 1.3	*2.7*	0	*0.3*	*0.5*	4	− 5.8	3.2	4	124.2	54.9	1	165.3	*11.6*	3	30.0	19.4
Taiga Shield	0	− *1.3*	*2.7*	3	0.3	0.5	0	− *5.8*	*3.2*	2	27.0	21.0	7	39.5	11.6	21	32.5	9.2
Total	37	− 4.9	1.3	9	− 1.8	1.8	20	− 4.3	1.4	62	35.8	6.2	24	43.7	8.2	71	39.8	6.2

Italic values indicate where values were missing and thus estimated from a similar ecozone

Daily average growing season NEE and CH_4_ emissions varied greatly within each peatland type (Table 3) and there was no statistically significant differences among peatland types. Daily NEE and CH_4_ emissions varied among ecozones for each peatland type, but sample sizes were too small to conduct statistical tests (Table 3). For bogs and both types of fen, NEE ranged from a strong sink (− 8.6 to − 12.5 g CO_2_ m^−2^ day^−1^) in Boreal Plains bogs and fens to a weak source (0.3 g CO_2_ m^−2^ day^−1^ and 0.4 g CO_2_ m^−2^ day^−1^) in Taiga Shield and Boreal Shield poor fens. However, some peatland types were represented by only a single or very few measurements in several ecozones. For CH_4_, emissions ranged from relatively small (0.9 mg CH_4_ m^−2^ day^−1^ from Boreal Plains poor fens) to large (> 100 mg CH_4_ m^−2^ day^−1^), with largest emissions from Taiga Plains for bogs (124.2 mg CH_4_ m^−2^ day^−1^) and poor fens (165.3 mg CH_4_ m^−2^ day^−1^). As with NEE, sample size was small for CH_4_ emissions for several of the ecozones. Combining ecozones into temperate (Atlantic Maritime and Mixedwood Plains), boreal (Boreal Plains and Boreal Shield) and subarctic (Hudson Plains, Taiga Plains and Taiga Shield) regions showed a trend of daily NEE from temperate < subarctic < boreal (p = 0.17). For CH_4_ emissions there was an interaction (p = 0.06) with region with bog < fen emissions for temperate (p = 0.03) and boreal regions (p = 0.04), and for fens subarctic < boreal < temperate (p = 0.05) (Table 4).

**Table 4:** Average and standard error of the mean (± SE) of daily growing season net ecosystem exchange (NEE) and methane (CH_4_) emissions for broad peatland regions of temperate (Atlantic Maritime and Mixedwood Plains), boreal (Boreal Shield and Boreal Plains), and subarctic (Hudson Plains, Taiga Shield and Taiga Plains)

Region	NEE (g CO_2_ m^−2^ day^−1^)			CH_4_ (mg CH_4_ m^−2^ day^−1^)		
	All peatlands	Bog	Fen	All peatlands	Bog	Fen
Temperate	− 2.0 (2.3)	− 2.7 (2.7)	− 1.3 (3.6)	48.2 (16.0)	30.6 (15.4)	65.8 (28.1)
Boreal	− 6.4 (1.3)	− 6.9 (1.5)	− 2.1 (2.2)	40.5 (5.7)	29.9 (8.6)	51.1 (7.5)
Subarctic	− 3.4 (1.8)	− 4.7 (3.0)	− 5.9 (1.9)	38.8 (6.4)	46.8 (10.6)	30.7 (7.0)

### Comparison of the PCM with the new peatlands map

The PCM [12, 13] and the newly created raster based peatlands map estimated different peatland areas (Table 5). The total bog and fen peatland area nationally was 11 × 10^5^ km^2^ for the PCM while the new peatlands map estimates an area of 7.3 × 10^5^ km^2^. In both maps the total peatland area for the seven dominant peatland-containing ecozones represented in this study (Atlantic Maritime, Mixedwood Plains, Boreal Shield, Boreal Plains, Hudson Plains, Taiga Shield and Taiga Plains) comprise 96% (for PCM) and 98% (for new peatlands map) of bog and fen peatland area in Canada (i.e., all 15 ecozones). The PCM had more bogs in the Taiga Plains, Taiga Shield, Boreal Shield, Atlantic Maritime, Mixedwood Plains and Hudson Plains than the new peatlands map, but the new peatlands map had more bogs in the Boreal Plains than the PCM. For fens, the outcome was the same with the PCM having greater areas of fens in all ecozones except the Boreal Plains. Both maps predicted a higher percentage of bogs than fens (66% bog, 34% fen for PCM; 73% bog, 27% fen for new peatlands map).

**Table 5:** Peatland areas by ecozone [69] from the Peatlands of Canada Map [13] and the new peatlands map from this study

Ecozone	Peatlands of Canada peatland areas (10^3^ km^2^)				New peatland map peatland areas (10^3^ km^2^)			New peatland map peatland areas by expanded categories (10^3^ km^2^)								
	Bog	Fen	Swamp	Marsh	Bog	Poor fen	Rich fen	Forested Bog	Treed Bog	Open Bog	Forested Poor Fen	Treed Poor Fen	Open Poor Fen	Forested Rich Fen	Treed Rich Fen	Open Rich Fen
Arctic Cordillera	0.1	0.0	0.0	0.0	0.0	0.0	0.0	0.0	0.0	0.0	0.0	0.0	0.0	0.0	0.0	0.0
Northern Arctic	2.0	2.5	0.0	0.0	0.0	0.0	0.0	0.0	0.0	0.0	0.0	0.0	0.0	0.0	0.0	0.0
Southern Arctic	13.3	3.1	0.0	0.0	0.0	0.0	0.0	0.0	0.0	0.0	0.0	0.0	0.0	0.0	0.0	0.0
Taiga Plains	*131.1*	*44.6*	*0.0*	*0.0*	*71.6*	*4.9*	*0.2*	*43.4*	*17.0*	*11.1*	*3.5*	*1.4*	*0.1*	*0.1*	*0.1*	*0.003*
Taiga Shield	*81.6*	*55.9*	*0.0*	*1.0*	*33.4*	*0.3*	*0.012*	*4.0*	*7.8*	*21.6*	*0.1*	*0.1*	*0.1*	*0.003*	*0.003*	*0.006*
Boreal Shield	*259.8*	*58.8*	*3.5*	*0.0*	*218.7*	*48.8*	*8.1*	*187.0*	*25.0*	*6.8*	*41.0*	*5.9*	*1.8*	*5.1*	*2.1*	*0.9*
Atlantic Maritime	*8.0*	*2.1*	*0.3*	*0.1*	*5.5*	*0.4*	*0.001*	*5.1*	*0.3*	*0.1*	*0.3*	*0.03*	*0.001*	*0.001*	*0.001*	*0.0*
Mixedwood Plains	*1.7*	*1.1*	*1.7*	*0.5*	*1.4*	*0.005*	*0.0*	*1.2*	*0.2*	*0.011*	*0.004*	*0.001*	*0.0*	*0.0*	*0.0*	*0.0*
Boreal Plains	*83.1*	*64.2*	*0.0*	*0.0*	*106.4*	*57.0*	*26.7*	*88.2*	*12.4*	*5.8*	*48.6*	*7.0*	*1.4*	*21.4*	*5.1*	*0.3*
Prairie	0.2	0.4	0.0	0.0	2.4	0.4	0.1	1.1	1.3	0.1	0.3	0.1	0.0	0.1	0.0	0.0
Taiga Cordillera	4.1	0.0	0.0	0.0	1.8	0.0	0.0	0.1	0.7	1.1	0.0	0.0	0.0	0.0	0.0	0.0
Boreal Cordillera	5.7	1.4	0.0	0.0	5.6	0.0	0.0	1.5	2.8	1.3	0.0	0.0	0.0	0.0	0.0	0.0
Pacific Maritime	3.4	0.3	0.0	0.0	0.1	0.0	0.0	0.0	0.0	0.1	0.0	0.0	0.0	0.0	0.0	0.0
Montane Cordillera	3.2	9.3	0.0	0.0	0.7	0.0	0.0	0.2	0.1	0.4	0.0	0.0	0.0	0.0	0.0	0.0
Hudson Plains	*127.7*	*130.4*	*0.0*	*0.2*	*85.5*	*34.9*	*13.7*	*33.5*	*19.2*	*32.8*	*17.4*	*13.0*	*4.5*	*3.2*	*8.4*	*2.1*
Total	724.9	374.1	5.5	1.9	533.1	146.7	48.9	365.2	86.7	81.2	111.4	27.6	7.8	29.8	15.7	3.3
% of total area	66	34	0	0	73	20	7	50	12	11	15	4	1	4	2	0

Italic ecozone names are the seven primary peatland-containing ecozones considered in this study

The percentage of polygons where the new peatland map and the PCM were in good agreement for proportion of peatland types within polygons was high (Table 6A with 79% of polygons predicting within 1 category (in 10% intervals) of the 1:1 line. Results for fens were better (Table 6B), with 89% of polygons predicting peatland type percentages within 1 category of the 1:1 line. Generally fen and bog categories matched between the two maps (i.e., bogs were found where there was no fens and vice versa) (Table 6C and D).

**Table 6:**
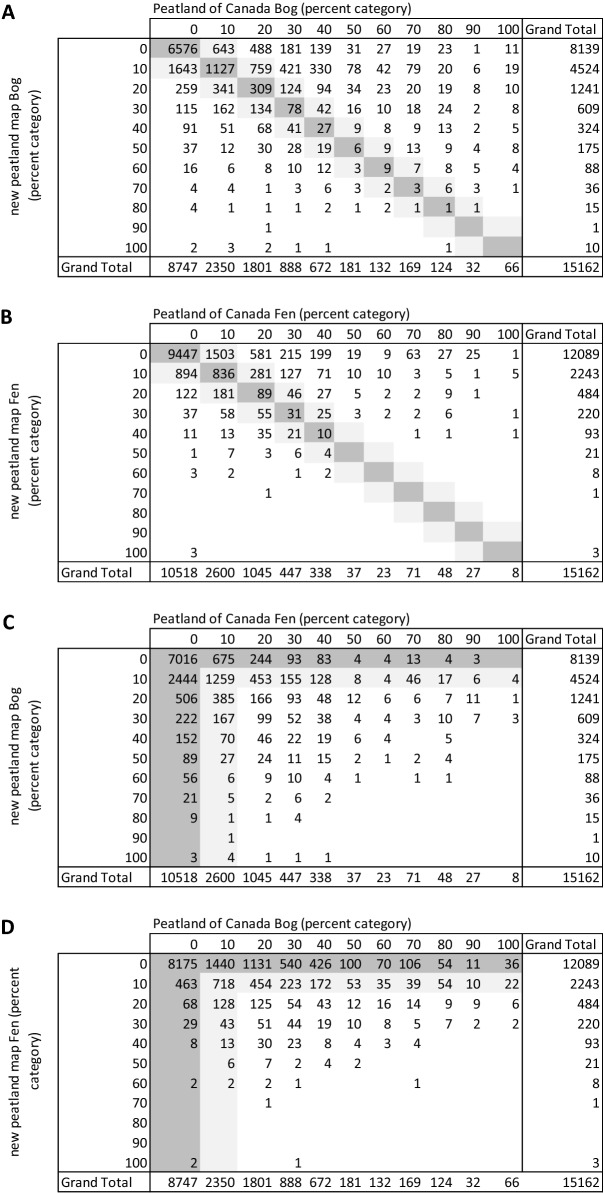
Comparison matrix for the number of peatland polygons in peatland type percentage categories from the Peatlands of Canada Map [13] and the new peatland map for: (A) bogs, (B) fens, and; (C) and (D) fens and bogs

The number of polygons where the percentage categories were the same in both mapping products are highlighted in dark gray, and where they differed by one category from where percentages were the same, are highlighted in light grey

Cross referencing the new peatlands map to geographic locations of the reported studies (data analysis not shown) showed that bogs were accurately identified as bogs. Sometimes forested or treed and open were confused although we often did not have enough information to evaluate the degree to which the study site was treed versus forested. Fens were less accurately identified, often being classified as bog. Even if accurately identified as fen, there was confusion between rich-intermediate and poor fen and open versus treed/forested fen. This validation is qualitative at best because geographic coordinates from the data source may have had rounding errors, and the projection system for the coordinates was unknown. Additionally, because the resolution of the k-NN map product is 250 m, differentiation of small peatland types would not be possible.

**Fig. 3:**
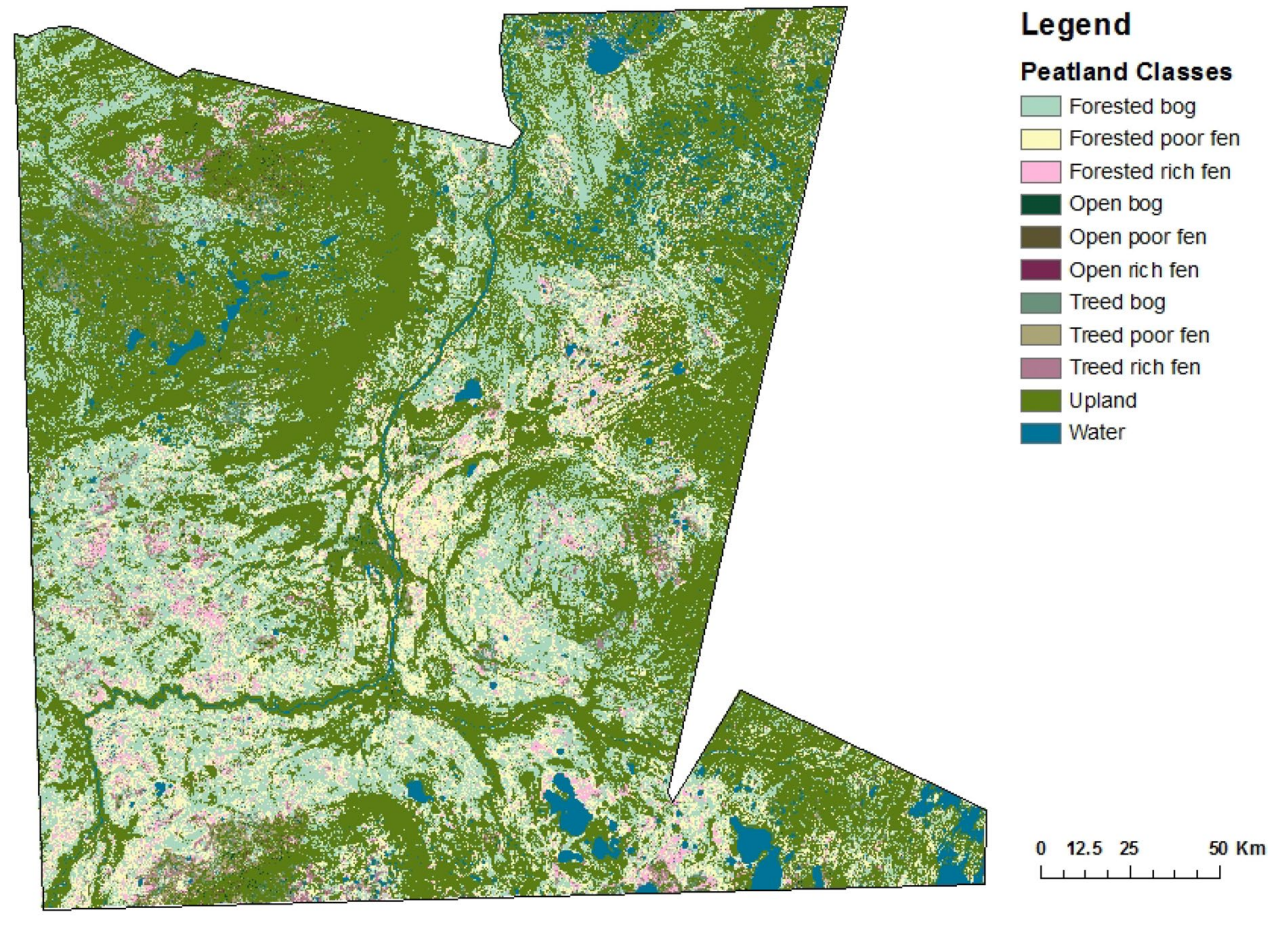
New peatlands map showing nine peatland types defined in Shaw et al. [44] for case study area in northern Alberta

Comparing the new peatlands map (Fig. 3) to the ground-truthed data collected by DUC for a portion of the Boreal Plains in northern Alberta (Table 7), showed an overall accuracy of 38%. There is some confusion with neighbouring peatlands on the gradient, but also some confusion between upland and peatland areas.

**Table 7:** Accuracy assessment of new peatlands map with the Ducks Unlimited Canada (DUC) wetland ground validation sites for case study area in northern Alberta

DU validation sites	New peatland map						
	Bog	Poor fen	Rich fen	Upland	Row total	Producer’s (%)	User’s (%)
Bog	64	4	21	0	89	38	72
Poor fen	34	3	29	0	66	30	5
Rich fen	12	0	41	0	53	37	77
Upland	58	3	19	0	80		
Column total	168	10	110	0	288		
Overall accuracy	38%						

Cells of the table show number of pixels for all the possible correlations between the ground truth (row) and the new peatland map (column). Overall accuracy is sum of diagonal pixels divided by total number of pixels. Errors of commission are described by the Producer’s accuracy, which is the percent correctly identified pixels within the column. Errors of commission are described by the User’s accuracy, which is the percent correctly identified pixels within the row. Overall kappa statistic is 0.11

### Scaling emissions

**Fig. 4:**
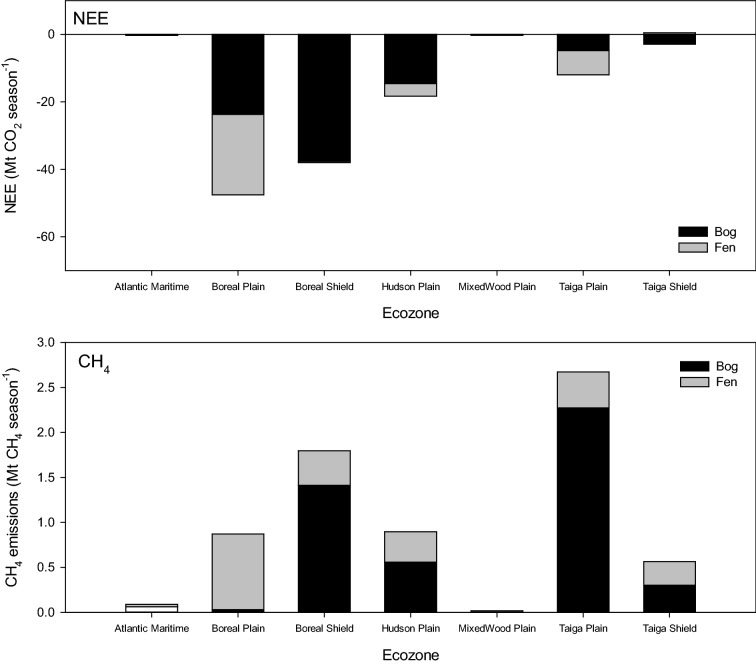
Ecozone [69] integrated seasonal net ecosystem exchange (NEE, Mt CO_2_ season^−1^, top panel) and methane (CH_4_, Mt CH_4_ season^−1^, bottom panel) emissions using the Peatland of Canada map [13]

**Fig. 5:**
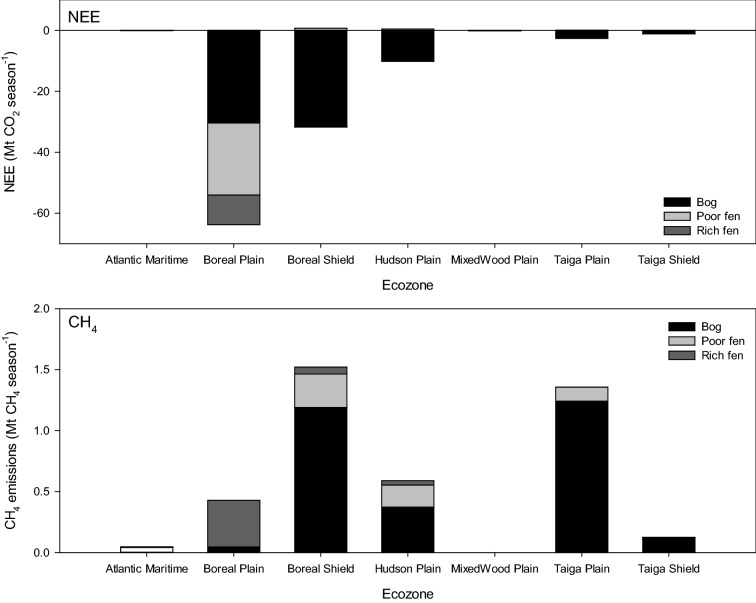
Ecozone [69] integrated seasonal net ecosystem exchange (NEE, Mt CO_2_ season^−1^, top panel) and methane (CH_4_, Mt CH_4_ season^−1^, bottom panel) emissions using the new peatlands map from this study

Using the PCM, NEE (± SE) for the seven major peatland-containing ecozones was calculated as a sink of -118.9 (66.4) Mt CO_2_ season^−1^ and CH_4_ as an emission of 6.9 (± 4.1) Mt CH_4_ season^−1^ (Table 8A). Using the new peatlands map, the sink was calculated as − 108.8 (± 41.3) Mt CO_2_ season^−1^ and CH_4_ as an emission of 4.1 (± 1.5) Mt CH_4_ season^−1^ (Table 8B). Converting CH_4_ to CO_2_e (multiplication by the global warming potential of 25 for CH_4_) resulted in a CO_2_e emission of 172.6 (101.4) Mt season^−1^ for CH_4_, and thus a net source for peatlands in Canada of 53.7 (167.7) Mt CO_2_e season^−1^ using the PCM. While using the new raster peatland map, the CH_4_ CO_2_e was 101.8 (36.4) Mt season^−1^ resulting in a net sink of − 7.0 (± 77.6) Mt CO_2_e season^−1^ for peatlands in Canada. Poor fens contribute most to the net source status being a moderate NEE sink but a small CO_2_e source. Bogs had large NEE but high CH_4_ resulting in a near neutral net emission (− 0.8 ± 63.8 Mt CO_2_e season^−1^) and rich fens were small sinks that were offset by CH_4_ emissions resulting in a small source (1.7 ± 6.1 Mt CO_2_e season^−1^). Using the PCM the Boreal Plains peatlands made the largest contribution to the national NEE sink (− 47.6 Mt CO_2_ season^−1^) and Taiga Plains peatlands contributed most to the national CH_4_ emissions (2.7 Mt CH_4_ season^−1^) (Fig. 4). Using the new raster peatlands map, Boreal Plains peatlands were also identified as the largest contribution to the NEE sink (− 63.8 Mt CO_2_ season^−1^) while and Boreal Shield peatlands contributed most to national CH_4_ emissions (1.5 Mt CH_4_ season^−1^) (Fig. 5).

**Table 8:** Scaled (average ± standard error) seasonal net ecosystem exchange (NEE, Mt CO_2_ season^−1^) and CH_4_ emissions (Mt CH_4_ season^−1^), CH_4_ as CO_2_ equivalents (Mt CO_2_e m^−2^ season^−1^) and net emission (Mt CO_2_e m^−2^ season^−1^) using

(A) Peatlands of Canada Map [13]						
Emission (Mt season^−1^)	Combined		Bog		Fen	
	Average	SE	Average	SE	Average	SE
NEE (CO_2_)	− 118.9	66.4	− 84.2	47.4	− 34.7	18.9
CH_4_	6.9	4.1	4.6	1.8	2.3	2.3
CH_4_ (CO_2_e)	172.6	101.4	116.0	44.8	56.6	56.6
Net emissions (CO_2_e)	53.7	167.7	31.8	92.2	21.9	75.5

(B) new peatland map								
Emission (Mt season^−1^)	Combined		Bog		Poor fen		Rich fen	
	Average	SE	Average	SE	Average	SE	Average	SE
NEE (CO_2_)	− 108.8	41.3	− 76.0	36.4	− 22.6	2.2	− 10.2	2.7
CH_4_	4.1	1.5	3.0	1.1	0.6	0.2	0.5	0.1
CH_4_ (CO_2_e)	101.8	36.4	75.3	27.4	14.6	5.6	11.9	3.4
Net emissions (CO_2_e)	− 7.0	77.6	− 0.8	63.8	− 7.9	7.7	1.7	6.1

For an annual estimate of emissions we assumed, based on the average of several studies that have quantified winter CO_2_ and CH_4_ emissions (Tables 1, 2), a non-growing season emission of 0.9 g CO_2_ m^−2^ day^−1^ and 7 mg CH_4_ m^−2^ day^−1^. By including non-growing season CO_2_ emissions, the peatlands switch from a sink of − 118.9 to a source of 80.2 Mt CO_2_ m^−2^ year^−1^ using the PCM, and from a sink of − 108.8 to a source of 24.2 Mt CO_2_ m^−2^ year^−1^ using the new peatlands map. Total net emissions including winter CH_4_ emissions increased from 53.7 to 291.5 Mt CO_2_e m^−2^ year^−1^ for PCM and from − 7.0 to 151.8 Mt CO_2_e m^−2^ year^−1^ for the new peatlands map.

### Potential climatic drivers of peatland NEE and CH_4_ emissions

**Fig. 6:**
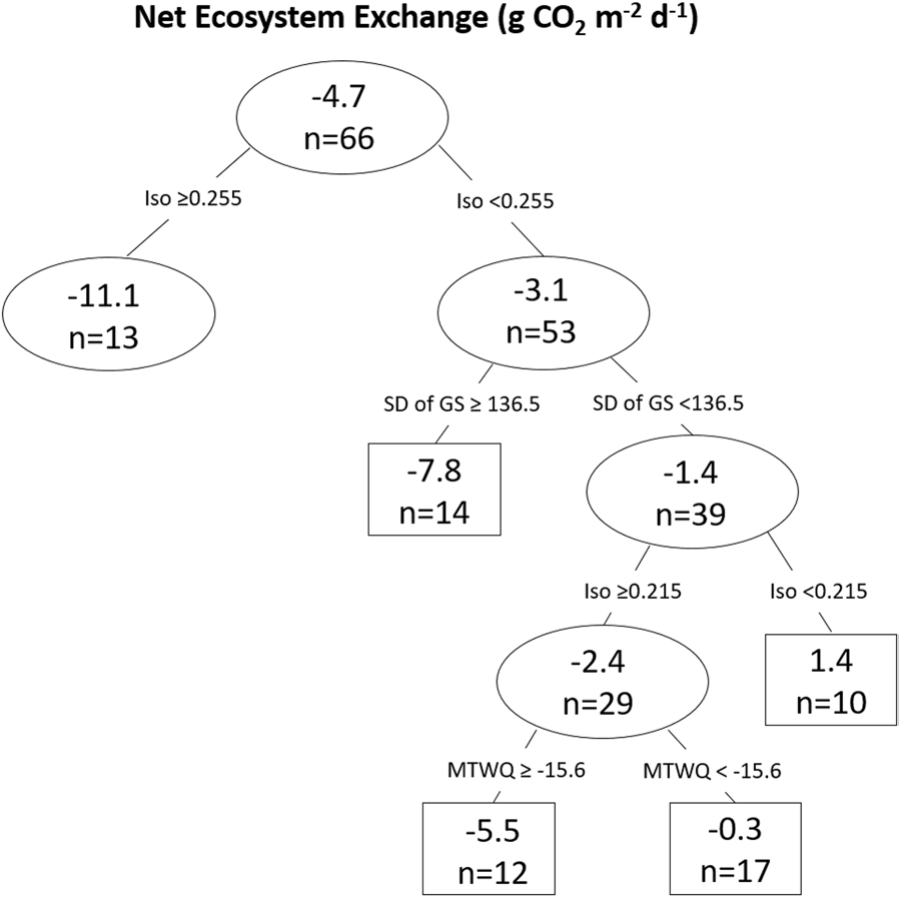
Regression tree predicting average daily net ecosystem exchange (g CO_2_ m^−2^ day^−1^) from peatlands from climate parameters (r^2^ = 0.40). Ovals represent intermediate nodes and mean value of number of observations within the node. Boxes are terminal nodes and mean value and number of observations within node. Climate factors and split values are indicated above the nodes. Iso is isothermality, SD of GS is start date of growing season, and MTWQ is mean temperature of warmest quarter of the year

**Fig. 7:**
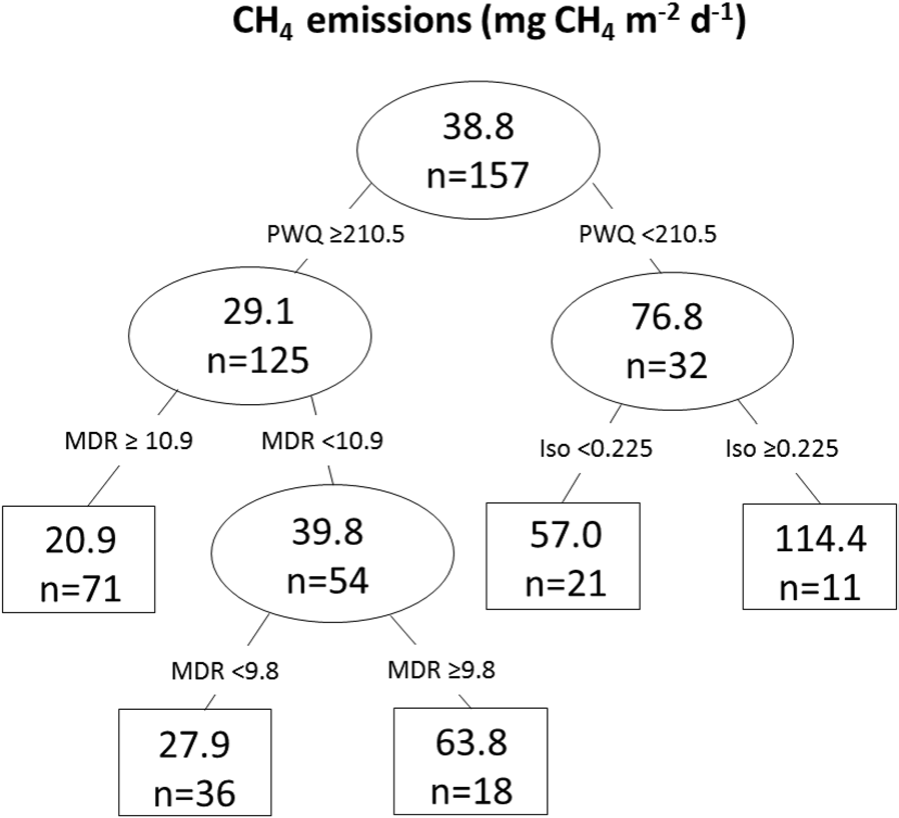
Regression tree for predicting average daily methane emissions (mg CH_4_ m^−2^ day^−1^) from peatlands from climate parameters (r^2^ = 0.29). Ovals represent intermediate nodes and mean value and number of observations within the node. Boxes are terminal nodes and mean value and number of observations within node. Climate factors and split values are indicated above the nodes. PWQ is precipitation of warmest quarter, MDR is mean diurnal range, and Iso is isothermality

Climate factors were slightly better at predicting daily average NEE (r^2^ = 0.40) than daily average CH_4_ emission (r^2^ = 0.29) in the regression trees. The regression tree identified temperature-related factors (isothermality, mean temperature of warmest quarter) along with start date of growing season as key to explaining variation in NEE (Fig. 6). Large negative NEE values (i.e., a sink) occurred where there was high temperature isothermality (≥ 0.255). This category with the largest sink contained primarily Boreal Plains bogs. Positive NEE values (i.e., a source) occurred where there was low temperature isothermality (< 0.255) and earlier start to growing season (< 136.5). This category was the largest source and contained primarily Boreal Shield bogs. The tree regression identified a precipitation-related factor (total precipitation in warmest quarter) and temperature parameters (mean diurnal range and isothermality) as key factors for explaining variation in CH_4_ emissions (Fig. 7). The highest CH_4_ emissions occurred from areas with low precipitation in warmest quarter (< 210.5 mm) and high temperature isothermality (≥ 0.225). The categories with the highest CH_4_ emissions were primarily the rich-intermediate fens of the Boreal Plains. Lowest CH_4_ emissions were found in regions with high precipitation in the warmest quarter (≥ 210.5 mm) and high mean diurnal range (≥ 10.9). The category with the lowest CH_4_ emissions was large and composed of bogs and fens from primarily the Hudson Plains and Boreal Shield.

## Discussion

### Scaled NEE and CH_4_ emissions

In previous studies average annual rates of C accumulation were assumed to be between 20 and 30 g C m^−2^ year^−1^ (equivalent to uptake of 73 to 110 g CO_2_ m^−2^ year^−1^) based on the average LORCA over the last six to eight thousand years [8, 14–17]. Based on the averages of compiled seasonal values and seasonal estimates predicted from daily values, we estimate average seasonal CO_2_ uptake across all studies at 171.6 (± 35.1) g CO_2_ m^−2^ season^−1^, which is higher than the previously reported range, although our estimate for average daily NEE of − 4.3 ± 7.1 g CO_2_ m^−2^ day^−1^ is similar to that reported by Lund et al. [26] for average daily rates of NEE in July of − 4.4 ± 3.2 g CO_2_ m^−2^ day^−1^ for 12 northern peatlands across North America and Europe. The seasonal CH_4_ emission rate across all studies (6.6 ± 0.7 g CH_4_ m^−2^ season^−1^) was within the range of 1 to 10 g CH_4_ m^−2^ year^−1^ cited by Moore and Knowles [53] and lower than the average for northern peatlands of 16 (standard deviation ± 28) g CH_4_ m^−2^ year^−1^ [54]. Our average daily rate for CH_4_ emission (38.8 mg CH_4_ m^−2^ day^−1^) was also lower than mean fluxes for temperate, boreal and subarctic regions (range of 72.7 to 112.2 mg CH_4_ m^−2^ day^−1^) reported by Turetsky et al. [55].

Using the seven major ecozone-specific seasonal emission rates for bogs and fens, we estimate a spatially-integrated seasonal NEE to be − 108.8 (± 41.3) Mt CO_2_ season^−1^ for the new peatlands map and − 118.9 (± 66.4) Mt CO_2_ season^−1^ for the PCM, which is a similar sink to previous national estimates of Kurz et al. [11] at 96.0 Mt CO_2_ year^−1^ and Roulet [6] at 91.6–135.6 Mt CO_2_ year^−1^. The new estimate for CH_4_ emissions from Canada’s peatlands at 4.1 ± 1.5 Mt CH_4_ season^−1^ using the new peatland map is larger than the estimate of Kurz et al. [11] at 0.8 Mt CH_4_ year^−1^ and within the range of Moore and Roulet [18] and Roulet [6] at 2–5 Mt CH_4_ y^−1^, although the PCM estimate at 6.9 (± 4.1) Mt CH_4_ season^−1^ is higher than this range.

The IPCC Wetlands Supplement provides GHG estimation guidance for international reporting of annual peatland fluxes. However, peatland GHG studies frequently do not include winter observations. It is difficult to assess winter emissions, as they are difficult to measure and they are typically assumed to be negligible. However, many have noted that these winter emissions may in fact not be negligible, particularly since snow covered soil can maintain temperatures much higher than the air temperature, allowing microbial metabolism to continue [56–60]. Using an approach that includes an estimate of winter emissions, the peatlands switched from a sink to a source of CO_2_ to the atmosphere. The daily rate of 0.9 g CO_2_ m^−2^ day^−1^ for the non-growing season used is larger than the average winter (January) emission rates assessed in northern peatlands across North America and Europe from eddy covariance studies of 0.5 ± 0.5 g CO_2_ m^−2^ day^−1^ [26]. At this lower emission rate, peatlands would be annual sinks and not sources. This analysis highlights uncertainties and knowledge gaps in understanding winter emissions which have a large effect when emissions are temporally and spatially scaled.

There are large standard errors on both NEE and CH_4_ emission estimates. The large errors in these estimates are due to small sample sizes in emission measurements in some ecozones that have large peatland areas (which magnify errors). Other key sources of error are spatial heterogeneity and temporal variability of emission within peatland types, among peatland types and across ecozones as well as error associated with estimating the total area of each peatland type. Furthermore, chamber measurements typically do not include larger shrubs and trees, thus NEE will be underestimated in the studies where these plant functional types are important. Despite the large uncertainty, this estimate improves on previous estimates by stratifying peatlands by peatland type and ecozone.

#### Spatial variability in emission rates

Peatland NEE and CH_4_ emissions were variable within peatland types. A single peatland is made up of many different microforms, including hummocks, lawns, hollows and shallow pools. It is difficult to generate the proportion of these microforms on a landscape scale. Instead, we accounted for this within-site variability by including microforms as different entries for each peatland type. Some of these features may cover a small area but produce disproportionately large fluxes. For example, NEE from small hummocks dominate CO_2_ uptake [61] while shallow pools have high CO_2_ emissions [60, 62]. For CH_4_, hummocks have lower emissions, followed by lawns and hollows having the highest emissions [63]. Permafrost (e.g., peat plateau or palsa) or recent thaw features (e.g., new collapse scars and shallow pools), which we could not account for, have even higher CH_4_ emissions [57, 64, 65]. Less recent permafrost thaw (e.g. collapse bogs) were included in our analysis which may confound trends for peatland types in subarctic areas. This fine-scale heterogeneity translated into large uncertainty in the estimates integrated across the seven ecozones.

Net ecosystem exchange and CH_4_ emissions also varied among peatland types. For NEE, the literature suggests that poor fens should be stronger CO_2_ sinks than rich fens [24, 25], however our synthesis did not support this observation. Vegetation strongly influences the gross ecosystem production (GEP) and ecosystem respiration (ER) components of NEE with both GEP and ER increasing from open to shrubby to wooded peatlands, correlating to aboveground vascular biomass [66]. It is possible the poor fens, which were under-represented in our analysis, may have been biased towards open systems which are weaker sinks. Additionally, chamber based measurements don’t sufficiently capture uptake of larger shrubs and trees, which would result in underestimation of the CO_2_ uptake. For CH_4_, emissions were extremely variable as have been observed in other northern peatlands [54]. Our results were consistent with the general trend noted in the literature that emissions typically decrease along the gradient of fen to bog [53] for the boreal and temperate ecozones, although this was not the case in the subarctic ecozones. For example, CH_4_ emissions from bogs were greater than CH_4_ emissions from fens in the Hudson Plains [67] and raised bogs and patterned poor fens can have high CH_4_ emissions where the peat mat is degrading [68]. There also may be a legacy effect in peatlands that have experienced permafrost thaw that could result in elevated CH_4_ emissions [65]. Vegetation can also modify this pattern among peatland types with treed peatlands having lower CH_4_ emissions than open bogs and fens [64], however our study could not quantify this effect.

Vegetation, instead of peatland type, may be more important for predicting emissions [55, 66]. However, the studies included in this analysis did not provide sufficient quantitative information on species composition and biomass to evaluate the effect of vegetation. For NEE, the biomass and leaf area index of vascular plants is important for GEP [25, 66]. For CH_4_, Bubier et al. [64] showed that bryophytes are a better predictor of emissions than vascular plants (e.g., shrubs), while sites with graminoids (e.g., *Carex* sp.) have also been found to be good predictors [55]. Shrub cover can indicate dry areas and low CH_4_ emissions, although this was only observed in subarctic fens, not in the boreal [64].

Net ecosystem exchange and CH_4_ emissions varied across ecozones. The NEE and CH_4_ trends by bog/fen and climate zone generally agree with values in the IPCC wetlands Supplement and other synthesis studies (Table 4). Previous work showed that among wetland regions [5], which are similar but not equivalent to ecozones [69], daily CO_2_ uptake at boreal and mid-latitude sites was greater than in the subarctic and arctic [56]. Our analysis is consistent with this observation with daily NEE highest in the boreal ecozones, followed by subarctic and temperate ecozones (p = 0.17). For CH_4_, previous work showed that emissions were lowest from Hudson Plains and Low Boreal followed by southern subarctic, northern subarctic, arctic and highest from the high boreal [18, 70]. However, Turetsky et al. [55] showed CH_4_ emissions were higher in temperate sites than subarctic and boreal. Our results did not show clear trends across regions when peatlands were combined, but the fen data followed the trend described by Turetsky et al. [55] (p = 0.05, Table 4). On a nationally integrated basis, Moore and Knowles [53] suggested the majority of the national CH_4_ flux came from subarctic wetland region fens, whereas our analysis suggests Boreal Shield and Taiga Plains peatlands contribute the most to these emissions. However, few emission measurements in the Taiga Plains combined with the large peatland area introduce large uncertainty in this estimate. Inversion modelling studies of Pickett-Heaps et al. [71] and Thompson et al. [72] have predicted emissions in the range of 2.3 to 3.4 Mt CH_4_ year^−1^ from the Hudson Plains. Our estimate for the Hudson Plain of 0.6 Mt CH_4_ season^−1^ (or 0.8 Mt CH_4_ year^−1^ including winter emissions) is smaller than these recent predictions, but close to an earlier inversion modelling prediction of Roulet et al. [67]: 0.5 Mt CH_4_ year^−1^]. However, the area used in the more recent inversion studies incorporates boreal and taiga areas outside the mapped Hudson Plains ecozone, which may contribute to higher estimates from inversion modelling. The limited sample size of ground measurements in the Hudson Plains is an additional source of uncertainty in this analysis. In addition, many studies based on chamber measurements may not fully capture CH_4_ emissions from ebullition, resulting in smaller emission rates compared to atmospheric concentrations.

#### Temporal variability in emission rates and the role of climate

Our analyses collapsed temporal variability in emissions. Average daily emissions over the growing season was the base unit for scaling and the meta-analysis included different years of measurements over the last 20–30 years. Some measurements were single measurements that may have reflected abnormal climate years. Where multi-year measurements were recorded the average of years was used to reduce the inter-annual variability. Climate conditions within a year are also known to affect emissions. Net ecosystem exchange decreases in dry, warm years and increases in warm and wet summers, reflecting the relative changes in photosynthesis and respiration [56, 59–61, 73]. A warmer, earlier snowmelt in spring and/or a warmer fall can lead to a net source of CO_2_ due to heterotrophic respiration increasing before photosynthesis begins, or heterotrophic respiration continuing after photosynthesis has stopped [74, 75]. The spring period following snowmelt has been shown, in particular, to have marked variation in NEE [74, 76]. Decreases in water table lower NEE, but increases in water table do not necessarily increase NEE if temperature and photosynthetically active radiation concurrently decrease [56]. For example, wetter conditions affect fens and bogs differently due to differences in the relative contributions of vascular plants and mosses [77]. Methane emissions are sensitive to water table and soil temperature [18] and tend to increase in warm, wet years [63]. This intra and inter-annual temporal variability can translate into large uncertainty in estimates at a national scale.

The regression tree identified potential bioclimatic drivers of the differences in daily emissions occurring at larger spatial scales. The regression tree included peatland type as a predictor but it was not identified as an explanatory variable. This may indicated that vegetation type is more important than peatland type. However, the regression trees did highlight bioclimatic variables such as temperature and moisture (precipitation) known to influence NEE and CH_4_ emissions. A more thorough analysis with detailed vegetation information related to species, cover and biomass, although not available for this analysis, would be more informative. Future studies should record such information when flux measurements are taken.

#### Improving national peatland maps

Despite improvements in understanding the processes driving NEE and CH_4_ emissions from peatlands our national maps of peatland distribution remain rather simplistic. While local and some regional scale high resolution peatland mapping has occurred (e.g., DUC 19 unit wetland classification; Smith undated) which has taken advantage of advances in remote sensing technology related to detecting peatlands and indicator species, efforts at the national scale have been absent until relatively recently (e.g., [45]). Efforts to stitch together provincial datasets (Canadian Wetland Inventory; http://maps.ducks.ca/cwi/) have proven challenging due to discrepancies in the peatland categories used. Typically these maps, similar to PCM, do not differentiate fen types, nor do they include percent tree cover. Thus the new peatlands map presented here, synthesized from existing national coverages, is an improvement over the polygon-based PCM.

Comparing the new map to the PCM shows a discrepancy in total peatland area, with the new map predicting lower peatland areas than the PCM, although both highlight the dominance of bogs as peatland type. The PCM includes other organic soil types such as Folisols that are not peatland soils, that may have contributed to overestimated peatland areas. The new peatlands map presented here underestimates the area of permafrost peatlands near the treeline, as low digital elevation model slopes were used to detect large flat areas as a predictor in peatland distribution [45]. However, in permafrost palsas, the 1–2 m of uplift surrounding non-permafrost wetlands creates higher slopes that are difficult to distinguish from upland areas. We recognize there are limitations to both the NFI k-NN map product and EOSD datasets that form the basis of our new map, that affected the map accuracy (Table 7), but as new and improved national data layers become available, this product can be quickly and continually refined. The new map, by being raster based and differentiating rich fen from poor fen is an improvement in predicting peatland distribution. The new map also has additional information about vegetation type (including open, treed and forested) and separation of studies into open, treed and forested peatlands, which has been suggested will improve predictions [55]. However, not enough detailed vegetation information was present in most data sources used in this study, thus differentiating woody vegetation influence within each ecozone was not possible.

### Climate change

In Canada, significant changes in temperature and precipitation have occurred since the beginning of the 20th century [78]. Significant changes are taking place in seasonal climatic pattern, which are likely to have major impacts on the NEE and CH_4_ seasonal emissions patterns for different peatland types [79–81]. Warmer temperatures will increase the growing season length. As a simple example, if the growing season length increases by 10% the sink strength of NEE increases (− 118.9 to − 130.8 Mt CO_2_ season^−1^ for PCM and − 108.9 to − 119.7 Mt CO_2_ season^−1^ for new peatland map). This is consistent with a longer growing season contributing to higher net ecosystem C sequestration [82]. However CH_4_ emissions also increase (4.7 to 5.2 Mt CH_4_ season^−1^ and 4.1 to 4.5 Mt CH_4_ season^−1^ for PCM and new peatland map respectively). For this scenario, the net emissions of CO_2_e increases from 57 to 63 Mt CO_2_e year^−1^ using the PCM, and decreases from − 7.0 to − 7.7 Mt CO_2_e year^−1^ using the new peatlands map. However, Moore and Roulet [18] suggest that climate change will likely decrease CH_4_ emissions due to CH_4_ production having a greater sensitivity to water table fluctuations than to temperature (i.e. growing degree days), so an increase in CO_2_e is not likely. The impact of climate change is likely to be even more complicated. Using peatland drainage as a substitute for water table decline with climate change, Strack et al. [83] found that with draining, respiration increased from hummocks, hollows and lawns, while GEP decreased in hummocks, but increased in lawns and hollows. Strack et al. [84] also showed that the response to climate change will depend on the antecedent moisture conditions of the site and succession. Over the long-term, persistent changes to climate and water table will also result in changes in plant communities. Invasion of vascular species with greater leaf area index will increase productivity thus greater photosynthesis will offset increases in respiration [61].

In areas where permafrost is currently present, accelerated thawing with climate change is expected to alter both NEE and CH_4_ emissions. Permafrost thaw creates wet internal lawns within drier bogs, which will increase respiration of CO_2_ by 1.6 times and CH_4_ emissions by 30 times [65]. Despite increases in respiration, there will be higher net C storage within biomass, however net storage will be partially, or completely, offset by increases in CH_4_ emissions for at least 70 years [85]. After 70–100 years, succession will allow peatlands to act like a thawed bog and be a small sink [86]. Another complicating factor of climate change is the expected change to fire frequencies within peatlands. A fire results in immediate loss of stored C and the peatland becomes a C source [87]. Weider et al. [88] found that immediately after fire boreal bogs in Alberta are a net source, then return to a sink 13 years post fire and reach peak strength at 75 years with a slight reduction in sink strength after 100 years.

## Conclusions

We present a synthesis of available estimates of NEE and CH_4_ emissions across Canada and use the PCM and a newly synthesized peatland map to calculate peatland emissions during the growing season from the seven major peatland-containing ecozones. This analysis is the best available to date, but highlights many uncertainties in up-scaling estimates. Combining data sets that used different methodologies, length of study and over different years is challenging. We attempted to maximize use of all data by using GS to standardize amongst daily, seasonal and annual reported values. While the spatial extent for which we have measurements within Canada has greatly increased in the last 20–30 years, some ecozones have few or no observational studies of NEE and CH_4_ emission in some peatland types. Assumptions had to be made for peatland types in ecozones where there were no studies. Future field studies need to be conducted in areas currently under-represented and even within well represented ecozones more consideration needs to be given to understanding the effect that vegetation differences (particularly the differences among open, treed and forested) have within peatland types. Similarly, microforms within peatlands can have large effects on emissions, but the proportion of the peatland which these landforms cover is often not quantified. Permafrost thaw features and shallow water pools are not explicitly captured in this analysis due to the difficulty in delineating them at a national scale, yet they are known to have large impacts on NEE and CH_4_ emissions.

In addition to uncertainties in emission rates, an added source of error is the incomplete knowledge of the distribution of peatland types within Canada. The Peatlands of Canada polygon-based map, was the best map possible given the technology of 20–30 years ago. Efforts to create a uniform layer or stitch together provincial raster maps of various qualities and containing different peatland types have been slow. We present an improved raster peatland map at 250 m resolution developed by synthesizing existing spatial information. Higher resolution maps containing many different peatland types (e.g., DUC enhance wetland classification) using an assortment of remote sensing technologies have only been prepared over small areas, and this is likely to be an area of continued development in the coming decade. Using remote sensing at a higher resolution will allow us to better predict the vegetation and thus identify key indicator species for peatland types and quantify woody biomass that will be key in improving emission estimates.

Climate was identified as an important driver of NEE and CH_4_ emissions. Temperature and precipitation influence microbial respiration and C turnover, permafrost melt, peat drying and fire susceptibility. Climate changes will be variable across ecozones, and thus it is difficult to predict how national emissions will change under a future climate. Over short time scales it is likely that emissions of CO_2_ and CH_4_ will increase with greater decomposition, permafrost thaw and fires, but over the long term may stabilize or decrease if peatlands are completely converted to forested systems (i.e., if all peat the peat burns to mineral soil).

The next step in improving national estimates of NEE and CH_4_ emissions is underway with the development of the Canadian Model for Peatlands (CaMP) [44]. The CaMP is intended to simulate C stock changes and emissions for 11 peatland categories over contemporary (1990 to present) and future (10–100 years ahead) time frames. The CaMP will be compatible with the newest modeling framework of the Carbon Budget Model of the Canadian Forest Sector (CBM-CFS; [89]) and is designed for application at multiple scales (site level to national level) and for spatially-referenced (polygon based) and spatially-explicit (raster based; ≥ 30 m resolution) modeling approaches.

## Additional file

**Additional file 1.** Listing of reported net ecosystem exchange and methane fluxes from Canadian peatlands.

## Abbreviations

ANOVA: analysis of variance
BOREAS: Boreal Ecosystem-Atmosphere Study
C: carbon
CaMP: Canadian Model for Peatlands
CBM-CFS: Carbon Budget Model-Canadian Forest Sector
CH_4_: methane
CO_2_: carbon dioxide
CO_2_e: carbon dioxide equivalent
DUC: Ducks Unlimited Canada
EC: eddy covariance
EOSD: Earth Observation for Sustainable Development of Forests
ER: ecosystem respiration
GEP: gross ecosystem production
GHG: greenhouse gas
GS: growing season
GWP: global warming potential
IPCC: Intergovernmental Panel on Climate Change
LORCA: long-term apparent rate of carbon accumulation
NEE: net ecosystem exchange
NFI: National Forest Inventory
NOWES: Northern Wetland Study
NRCan: Natural Resource Canada
PCM: Peatlands of Canada Map
SLC: Soil Landscapes of Canada

## References

[1] Joosten H, Clarke D (2002). Wise use of mires and peatlands—background principles including a framework for decision-making.

[2] Waddington JM, Morris PJ, Kettridge N, Granath G, Thompson DK, Moore PA (2015). Hydrological feedbacks in northern peatlands. Ecohydrology.

[3] Apps MJ, Kurz WA, Luxmoore RJ, Nilsson LO, Sedjo RA, Schmidt R, Simpson LG, Vinson TS (1993). Boreal forests and tundra. Water Air Soil Pollut.

[4] Tarnocai C (2006). The effect of climate change on carbon in Canadian peatlands. Glob Planet Change..

[5] Zoltai SC. An outline of the wetland regions of Canada. In: Proceedings of the Canadian wetlands workshop. Environment Canada, Lands Directorate, Ecological Land Classification, vol 12; 1979. p. 1–8.

[6] Roulet NT (2000). Peatlands, carbon storage, greenhouse gases, and the Kyoto protocol: prospects and significance for Canada. Wetlands.

[7] Vitt DH, Wieder RK, Vitt DH (2006). Functional characteristics and indicators of boreal peatlands. Boreal peatland ecosystems. Ecological studies (analysis and synthesis).

[8] Gorham E (1991). Northern peatlands: role in the carbon budget and probable responses to global warming. Ecol Appl.

[9] Dimitrov DD, Grant RF, Lafleur PM, Humphreys ER (2011). Modeling the effects of hydrology on gross primary productivity and net ecosystem productivity at Mer Bleue bog. J Geophy Res Biogeosci.

[10] Intergovernmental Panel on Climate Change (IPCC). Supplement to the 2006 Guidelines for National Greenhouse Gas Inventories: Wetlands (Wetlands Supplement); 2014. https://www.ipcc-nggip.iges.or.jp/public/wetlands/. Accessed 16 Mar 2015.

[11] Kurz WA, Apps MJ, Webb TM, McNamee PJ. The carbon budget of the Canadian forest sector: Phase 1. Information Report NOR-X-326. Forestry Canada, Northwest Region, Northern Forestry Centre. Edmonton, Alberta; 1992.

[12] Tarnocai C, Kettles IM, Lacelle B. Peatlands of Canada; Geological Survey of Canada, Open File 3834, 1 CD ROM; 2000.

[13] Tarnocai C, Kettles IM, Lacelle B. Peatlands of Canada. Agriculture and Agri-Food Canada, Research Branch, Ottawa, (digital database); 2005.

[14] Gorham E, Janssens J, Glaser P (2003). Rates of peat accumulation during the postglacial period in 32 sites from Alaska to Newfoundland, with special emphasis on northern Minnesota. Can J Bot.

[15] Turunen J, Roulet NT, Moore TR, Richard PJH (2004). Nitrogen deposition and increased carbon accumulation in ombrotrophic peatlands in eastern Canada. Glob Biogeochem Cycles.

[16] Turunen J, Tomppo E, Tolonen K, Reinikainen A (2002). Estimating carbon accumulation rates of undrained mires in Finland—application to boreal and subarctic regions. Holocene.

[17] Vitt DH, Halsey A, Bauer IE, Campbell C (2000). Spatial and temporal trends in carbon storage of peatlands of continental western Canada through the Holocene. Can J Earth Sci.

[18] Moore TR, Roulet NT, Lal R, Kimble J, Levine E, Stewart BA (1995). Methane emissions from Canadian peatlands. Soils and global change.

[19] National Soil Database. Soil landscapes of Canada, v.2.2; Research Branch, Agriculture and Agri-Food Canada, Ottawa. (digital database); 1996.

[20] Soil Classification Working Group. The Canadian system of soil classification, 3rd Edn. Agriculture and Agri-Food Canada Publication 1646 (Revised); 1998. p. 187.

[21] Tarnocai C, Kettles IM, Lacelle B. Geological Survey of Canada, Open File 6561. Natural Resources Canada; 2011. https://geoscan.nrcan.gc.ca/starweb/geoscan/servlet.starweb?path=geoscan/fulle.web&search1=R=288786. Accessed 13 Aug 2012.

[22] Tarnocai C, Kroetsch S, Kroetsch D. Soil climates of the Mackenzie Valley (1994 progress report). Centre for Land and Biological Resources Research, Research Branch, Agriculture Canada, Ottawa; 1995.

[23] Melton JR, Wania R, Hodson EL, Poulter B, Ringeval B, Spahni R (2013). Present state of global wetland extent and wetland methane modelling: conclusions from a model intercomparison project (WETCHIMP). Biogeosciences.

[24] Adkinson AC, Syed KH, Flanagan LB (2011). Contrasting responses of growing season ecosystem CO_2_ exchange to variation in temperature and water table depth in two peatlands in northern Alberta, Canada. J Geophys Res Biogeosci.

[25] Glenn AJ, Flanagan LB, Syed KH, Carlson PJ (2006). Comparison of net ecosystem CO_2_ exchange in two peatlands in western Canada with contrasting dominant vegetation, Sphagnum and Carex. Agric For Meteorol.

[26] Lund M, Lafleur P, Roulet NT, Lindroth A, Christensen T, Aurela M, Chojnicki B, Flanagan L, Humphreys E, Laurila T, Oechel W, Olejnik J, Rinne J, Schubert P, Nilsson M (2010). Variability in exchange of CO_2_ across 12 northern peatland and tundra sites. Glob Change Biol.

[27] Olefeldt D, Turetsky MR, Crill PM, McGuire AD (2013). Environmental and physical controls on northern terrestrial methane emissions across permafrost zones. Glob Change Biol.

[28] Glooschenko WA, Roulet NT, Barrie LA, Schiff HI, McAdie HG (1994). The northern wetlands study (NOWES): an overview. J Geophys Res Atmos.

[29] Margolis HA, Flanagan LB, Amiro BD (2006). The Fluxnet-Canada Research Network: influence of climate and disturbance on carbon cycling in forests and peatlands. Agric For Meteorol.

[30] Sellers P, Hall F, Ranson KJ, Margolis H, Kelly B, Baldocchi D, den Hartog G, Cihlar J, Ryan MG, Goodison B, Crill P (1995). The boreal ecosystem-atmosphere study (BOREAS): an overview and early results from the 1994 field year. Bull Am Meteorol Soc.

[31] Altor AE, Mitsch WJ (2008). Methane emissions and carbon dioxide fluxes in created wetland mesocosms: effects of hydrologic regime and hydric soils. Ecol Appl.

[32] Couwenberg J, Thiele A, Tanneberger F, Augustin J, Barisch S, Dubovik D, Liashchynskaya N, Michaelis D, Minke M, Skuratovich A, Joosten H (2011). Assessing greenhouse gas emissions from peatlands using vegetation as a proxy. Hydrobiologia.

[33] Hargreaves KJ, Fowler D (1998). Quantifying the effects of water table and soil temperature on the emission of methane from peat wetland at the field scale. Atmos Environ.

[34] Peichl M, Öquist M, Löfvenius MO, Ilstedt U, Sagerfors J, Grelle A, Lindroth A, Nilsson MB (2014). A 12-year record reveals pre-growing season temperature and water table level threshold effects on the net carbon dioxide exchange in a boreal fen. Environ Res Lett.

[35] Bridgham SD, Richardson CJ (1993). Hydrology and nutrient gradients in North Carolina peatlands. Wetlands.

[36] Inglett KS, Inglett PW, Reddy KR, Osborne TZ (2012). Temperature sensitivity of greenhouse gas production in wetland soils of different vegetation. Biogeochemistry.

[37] Bartlett KB, Bartlett DS, Harriss RC, Sebacher DI (1987). Methane emissions along a salt marsh salinity gradient. Biogeochemistry.

[38] Bridgham SD, Pastor J, Dewey B, Weltzin JF, Updegraff K (2008). Rapid carbon response of peatlands to climate change. Ecology.

[39] Weston NB, Vile MA, Neubauer SC, Velinsky DJ (2011). Accelerated microbial organic matter mineralization following saltwater intrusion into tidal freshwater marsh soils. Biogeochemistry.

[40] Kandel TP, Elsgaard L, Lærke PE (2013). Measurement and modelling of CO2 flux from a drained fen peatland cultivated with reed canary grass and spring barley. Glob Change Biol Bioenergy.

[41] Mackey BG, McKenney DW, Yang YQ, McMahon JP, Hutchinson MF (1996). Site regions revisited: a climatic analysis of Hills’ site regions for the province of Ontario using a parametric method. Can J For Res.

[42] McKenney DW, Hutchinson MF, Papadopol P, Lawrence K, Pedlar J, Campbell K, Milewska E, Hopkinson R, Price D, Owen T (2011). Customized spatial climate models for North America. Bull Am Meteorol Soc.

[43] Systat Software, Inc (2011). SigmaPlot, Version 12 for Windows.

[44] Shaw CH, Bona KA, Thompson DK, Dimitrov DD, Bhatti JS, Hilger AB, Webster KL, Kurz WA. Canadian model for peatlands version 1.0: a model design document. Natural Resources Canada, Canadian Forest Service, Northern Forestry Centre, Edmonton, Alberta. Information Report NOR-X-425; 2016.

[45] Thompson DK, Simpson BN, Beaudoin A (2016). Using forest structure to predict the distribution of treed boreal peatlands in Canada. For Ecol Manage.

[46] Beaudoin A, Bernier PY, Villemaire P, Guindon L, Guo XJ (2017). Tracking forest attributes across Canada between 2001 and 2011 using ak nearest neighbors mapping approach applied to MODIS imagery. Can J For Res.

[47] Hijmans RJ, Philips S, Leathwick J, Elith J. Package ‘‘dismo”; 2015. http://cran.r-project.org/web/packages/dismo/dismo.pdf. Accessed 20 Nov 2015.

[48] Natural Resources Canada (NRCan). CanVec_50K_Hydro_shapefile; 2016. ftp.geogratis.gc.ca/pub/nrcan_rncan/vector/canvec/shp/Hydro/. Accessed Sept 2016.

[49] Wulder MA, White JC, Cranny M, Hall RJ, Luther JE, Beaudoin A, Goodenough DG, Dechka JA (2008). Monitoring Canada’s forests. Part 1: completion of the EOSD land cover project. Can J Remote Sens.

[50] Glaser PH, Janssens JA, Siegel DI (1990). The response of vegetation to chemical and hydrological gradients in the Lost River peatland, northern Minnesota. J Ecol.

[51] Slack NG, Vitt DH, Horton DG (1980). Vegetation gradients of minerotrophically rich fens in western Alberta. Can J Bot.

[52] R Core Team. R: A language and environment for statistical computing. R Foundation for Statistical Computing, Vienna, Austria; 2013. http://www.R-project.org/. Accessed 21 Nov 2017.

[53] Moore TR, Knowles R (1990). Methane emissions from fen, bog and swamp peatlands in Quebec. Biogeochemistry.

[54] Abdalla M, Hastings A, Truu J, Espenberg M, Mander Ü, Smith P (2016). Emissions of methane from northern peatlands: a review of management impacts and implications for future management options. Ecol Evol.

[55] Turetsky MR, Kotowska A, Bubier J, Dise NB, Crill P, Hornibrook ER, Minkkinen K, Moore TR, Myers-Smith IH, Nykänen H, Olefeldt D (2014). A synthesis of methane emissions from 71 northern, temperate, and subtropical wetlands. Glob Change Biol.

[56] Lafleur PM, Roulet NT, Bubier JL, Frolking S, Moore TR (2003). Interannual variability in the peatland-atmosphere carbon dioxide exchange at an ombrotrophic bog. Glob Biogeochem Cycles.

[57] Pelletier L, Moore TR, Roulet NT, Garneau M, Beaulieu-Audy V (2007). Methane fluxes from three peatlands in the La Grande Riviere watershed, James Bay lowland, Canada. J Geophys Res Biogeosci.

[58] Roehm CL, Roulet NT (2003). Seasonal contribution of CO_2_ fluxes in the annual C budget of a northern bog. Glob Biogeochem Cycles.

[59] Rouse WR, Bello RL, D’Souza A, Griffis TJ, Lafleur PM (2002). The annual carbon budget for fen and forest in a wetland at Arctic treeline. Arctic.

[60] Trudeau NC, Garneau M, Pelletier L (2014). Interannual variability in the CO_2_ balance of a boreal patterned fen, James Bay, Canada. Biogeochemistry.

[61] Griffis TJ, Rouse WR, Waddington JM (2000). Scaling net ecosystem CO2 exchange from the community to landscape-level at a subarctic fen. Glob Change Biol.

[62] McEnroe NA, Roulet NT, Moore TR, Garneau M (2009). Do pool surface area and depth control CO_2_ and CH_4_ fluxes from an ombrotrophic raised bog, James Bay, Canada?. J Geophys Res Biogeosci.

[63] Bubier J, Moore T, Savage K, Crill P (2005). A comparison of methane flux in a boreal landscape between a dry and a wet year. Glob Biogeochem Cycles.

[64] Bubier JL, Moore TR, Bellisario L, Comer NT, Crill PM (1995). Ecological controls on methane emissions from a northern peatland complex in the zone of discontinuous permafrost, Manitoba, Canada. Glob Biogeochem Cycles.

[65] Turetsky MR, Wieder RK, Vitt DH (2002). Boreal peatland C fluxes under varying permafrost regimes. Soil Biol Biochem.

[66] Humphreys ER, Lafleur PM, Flanagan LB, Hedstrom N, Syed KH, Glenn AJ, Granger R (2006). Summer carbon dioxide and water vapor fluxes across a range of northern peatlands. J Geophys Res Biogeosci.

[67] Roulet NT, Jano A, Kelly CA, Klinger LF, Moore TR, Protz R, Ritter J, Rouse WR (1994). Role of the Hudson Bay lowland as a source of atmospheric methane. J Geophys Res Atmos.

[68] Bubier JL (1995). The relationship of vegetation to methane emission and hydrochemical gradients in northern peatlands. J Ecol.

[69] Ecological Stratification Working Group. A National Ecological Framework for Canada. Agriculture and Agri-Food Canada, Research Branch, Centre for Land and Biological Resources Research and Environment Canada, State of the Environment Directorate, Ecozone Analysis Branch, Ottawa/Hull. Report and national map at 1:7500 000 scale; 1995.

[70] Moore TR, Heyes A, Roulet NT (1994). Methane emissions from wetlands, southern Hudson Bay lowland. J Geophys Res Atmos.

[71] Pickett-Heaps CA, Jacob DJ, Wecht KJ, Kort EA, Wofsy SC, Diskin GS, Worthy DEJ, Kaplan JO, Bey I, Drevet J (2011). Magnitude and seasonality of wetland methane emissions from the Hudson Bay Lowlands (Canada). Atmos Chem Phys.

[72] Thompson RL, Sasakawa M, Machida T, Aalto T, Worthy D, Lavric JV, Lund Myhre C, Stohl A (2017). Methane fluxes in the high northern latitudes for 2005–2013 estimated using a Bayesian atmospheric inversion. Atmos Chem Phys.

[73] Cai T, Flanagan LB, Syed KH (2010). Warmer and drier conditions stimulate respiration more than photosynthesis in a boreal peatland ecosystem: analysis of automatic chambers and eddy covariance measurements. Plant Cell Environ.

[74] Griffis TJ, Rouse WR, Waddington JM (2000). Interannual variability of net ecosystem CO_2_ exchange at a subarctic fen. Glob Biogeochem Cycles.

[75] Joiner DW, Lafleur PM, McCaughey JH, Bartlett PA (1999). Interannual variability in carbon dioxide exchanges at a boreal wetland in the BOREAS northern study area. J Geophys Res Atmos.

[76] Lafleur PM, Roulet NT, Admiral SW (2001). Annual cycle of CO_2_ exchange at a bog peatland. J Geophys Res Atmos.

[77] Sulman BN, Desai AR, Saliendra NZ, Lafleur PM, Flanagan LB, Sonnentag O, Mackay DS, Barr AG, van der Kamp G (2010). CO2 fluxes at northern fens and bogs have opposite responses to inter-annual fluctuations in water table. Geophys Res Lett.

[78] Price DT, Alfaro RI, Brown KJ, Flannigan MD, Fleming RA (2013). Anticipating the consequences of climate change for Canada’s boreal forest ecosystems. Environ Rev.

[79] Dinsmore KJ, Billett MF, Skiba U, Rees RM, Drewer J, Helfter C (2010). Role of the aquatic pathway in the carbon and greenhouse gas budgets of a peatland catchment. Glob Change Biol.

[80] Nilsson M, Sagerfors J, Buffam I, Laudon H, Eriksson T, Grelle A, Klemedtsson L, Weslien P, Lindroth A (2008). Contemporary carbon accumulation in a boreal oligotrophic minerogenic mire—a significant sink after accounting for all C-fluxes. Glob Change Biol.

[81] Roulet NT, Lafleur PM, Richard PJH, Moore TR, Humphreys ER, Bubier J (2007). Contemporary carbon balance and late Holocene carbon accumulation in a northern peatland. Glob Change Biol.

[82] Syed KH, Flanagan LB, Carlson PJ, Glenn AJ, Van Gaalen KE (2006). Environmental control of net ecosystem CO_2_ exchange in a treed, moderately rich fen in northern Alberta. Agric For Meteorol.

[83] Strack M, Waddington JM, Rochefort L, Tuittila ES (2006). Response of vegetation and net ecosystem carbon dioxide exchange at different peatland microforms following water table drawdown. J Geophys Res Biogeosci.

[84] Strack M, Waddington JM, Tuittila ES (2004). Effect of water table drawdown on northern peatland methane dynamics: implications for climate change. Glob Biogeochem Cycles.

[85] Robinson SD, Moore TR (1999). Carbon and peat accumulation over the past 1200 years in a landscape with discontinuous permafrost, northwestern Canada. Glob Biogeochem Cycles.

[86] Turetsky MR, Wieder RK, Vitt DH, Evans RJ, Scott KD (2007). The disappearance of relict permafrost in boreal North America: effects on peatland carbon storage and fluxes. Glob Change Biol.

[87] Turetsky M, Wieder K, Halsey L, Vitt D (2002). Current disturbance and the diminishing peatland carbon sink. Geophys Res Lett.

[88] Wieder RK, Scott KD, Kamminga K, Vile MA, Vitt DH, Bone T, Xu BIN, Benscoter BW, Bhatti JS (2009). Postfire carbon balance in boreal bogs of Alberta, Canada. Glob Change Biology.

[89] Kurz WA, Dymond CC, White TM, Stinson G, Shaw CH, Rampley GJ, Smyth C, Simpson BN, Neilson ET, Trofymow JA, Metsaranta J (2009). CBM-CFS3: a model of carbon-dynamics in forestry and land-use change implementing IPCC standards. Ecol Model.

[90] Strack M, Zuback YCA (2013). Annual carbon balance of a peatland 10 year following restoration. Biogeosciences.

[91] Wang M, Wu J, Lafleur PM, Luan J, Chen H, Zhu X (2018). Can abandoned peatland pasture sequestrate more carbon dioxide from the atmosphere than an adjacent pristine bog in Newfoundland, Canada?. Agric For Meteorol.

[92] Trudeau NC, Garneau M, Pelletier L (2013). Methane fluxes from a patterned fen of the northeastern part of the La Grande river watershed, James Bay, Canada. Biogeochemistry.

